# A meta-analysis of genome-wide studies of resilience in the German population

**DOI:** 10.1038/s41380-024-02688-1

**Published:** 2024-08-08

**Authors:** Marisol Herrera-Rivero, Linda Garvert, Katrin Horn, Margrit Löbner, Elena Caroline Weitzel, Monika Stoll, Peter Lichtner, Henning Teismann, Alexander Teumer, Sandra Van der Auwera, Henry Völzke, Uwe Völker, Till F. M. Andlauer, Susanne Meinert, Stefanie Heilmann-Heimbach, Andreas J. Forstner, Fabian Streit, Stephanie H. Witt, Tilo Kircher, Udo Dannlowski, Markus Scholz, Steffi G. Riedel-Heller, Hans J. Grabe, Bernhard T. Baune, Klaus Berger

**Affiliations:** 1 Department of Psychiatry, University of Münster, Münster, Germany; 2 Department of Genetic Epidemiology, Institute of Human Genetics, University of Münster, Münster, Germany; 3 Joint Institute for Individualisation in a Changing Environment (JICE), University of Münster and Bielefeld University, Münster, Germany; 4 Department of Psychiatry and Psychotherapy, University Medicine Greifswald, Greifswald, Germany; 5 Institute for Medical Informatics, Statistics and Epidemiology, Medical Faculty, University of Leipzig, Leipzig, Germany; 6 LIFE Research Center for Civilization Diseases, Medical Faculty, University of Leipzig, Leipzig, Germany; 7 Institute of Social Medicine, Occupational Health and Public Health (ISAP), Medical Faculty, University of Leipzig, Leipzig, Germany; 8 Department of Biochemistry, Genetic Epidemiology and Statistical Genetics, Maastricht University, Maastricht, Netherlands; 9 Core Facility Genomics, Helmholtz Centre Munich, Munich, Germany; 10 Institute of Epidemiology and Social Medicine, University of Münster, Münster, Germany; 11 German Centre for Cardiovascular Research (DZHK), Partner Site Greifswald, Greifswald, Germany; 12 German Center for Neurodegenerative Diseases (DZNE), Site Rostock/Greifswald, Greifswald, Germany; 13 Institute for Community Medicine, University Medicine Greifswald, Greifswald, Germany; 14 Interfaculty Institute of Genetics and Functional Genomics, University Medicine Greifswald, Greifswald, Germany; 15 Department of Neurology, Klinikum rechts der Isar, School of Medicine, Technical University of Munich, Munich, Germany; 16 Institute for Translational Psychiatry, University of Münster, Münster, Germany; 17 Institute of Human Genetics, University of Bonn, School of Medicine & University Hospital Bonn, Bonn, Germany; 18 Institute of Neuroscience and Medicine (INM-1), Research Center Jülich, Jülich, Germany; 19 Department of Genetic Epidemiology in Psychiatry, Central Institute of Mental Health, Medical Faculty Mannheim, Heidelberg University, Mannheim, Germany; 20 Department of Psychiatry and Psychotherapy, Central Institute of Mental Health, Medical Faculty Mannheim, Heidelberg University, Mannheim, Germany; 21 Hector Institute for Artificial Intelligence in Psychiatry, Central Institute of Mental Health, Medical Faculty Mannheim, Heidelberg University, Mannheim, Germany; 22 German Center for Mental Health (DZPG), partner site Mannheim/Heidelberg/Ulm, Ulm, Germany; 23 Department of Psychiatry and Psychotherapy, University of Marburg, Marburg, Germany; 24 Department of Psychiatry, Melbourne Medical School, The University of Melbourne, Melbourne, Australia; 25 The Florey Institute of Neuroscience and Mental Health, The University of Melbourne, Parkville, VIC Australia

**Keywords:** Genetics, Psychology, Psychiatric disorders

## Abstract

Resilience is the capacity to adapt to stressful life events. As such, this trait is associated with physical and mental functions and conditions. Here, we aimed to identify the genetic factors contributing to shape resilience. We performed variant- and gene-based meta-analyses of genome-wide association studies from six German cohorts (N = 15822) using the 11-item version of the Resilience Scale (RS-11) as outcome measure. Variant- and gene-level results were combined to explore the biological context using network analysis. In addition, we conducted tests of correlation between RS-11 and the polygenic scores (PGSs) for 12 personality and mental health traits in one of these cohorts (PROCAM-2, N = 3879). The variant-based analysis found no signals associated with resilience at the genome-wide level (p < 5 × 10^−8^), but suggested five genomic loci (p < 1 × 10^−5^). The gene-based analysis identified three genes (*ROBO1*, *CIB3* and *LYPD4*) associated with resilience at genome-wide level (p < 2.48 × 10^−6^) and 32 potential candidates (p < 1 × 10^−4^). Network analysis revealed enrichment of biological pathways related to neuronal proliferation and differentiation, synaptic organization, immune responses and vascular homeostasis. We also found significant correlations (FDR < 0.05) between RS-11 and the PGSs for neuroticism and general happiness. Overall, our observations suggest low heritability of resilience. Large, international efforts will be required to uncover the genetic factors that contribute to shape trait resilience. Nevertheless, as the largest investigation of the genetics of resilience in general population to date, our study already offers valuable insights into the biology potentially underlying resilience and resilience’s relationship with other personality traits and mental health.

## Introduction

Resilience refers to the ability of an individual to adapt to and recover from stressful or difficult living situations and conditions [1]. Thus, resilience can be conceptualized as a trait, an outcome or a process. As opposed to the latter, trait resilience is a relatively stable characteristic of an individual’s personality [2]. However, it has been shown that trait resilience correlates with mental health indicators in the presence of adversity [1]. Because the level of resilience of each individual is thought to derive from an interaction between risk and protective factors, such as stress and social support, respectively [3], it becomes crucial to identify these factors in order to improve our understanding of psychological conditions and enable resilience-based interventions that promote mental health.

Various studies have associated trait resilience with positive and negative indicators of mental health, such as life satisfaction, positive affect, depression and anxiety, as well as with daily life well-being and other personality traits, including the Big Five (neuroticism, extraversion, openness, agreeableness and conscientiousness) [1, 2, 4]. Similar to other personality characteristics, resilience can be measured using different scales developed for this purpose, including the Resilience Scale [3, 4]. Such instruments have enabled the study of the genetic architecture of various personality traits, uncovering moderate polygenic contributions to both personality and psychopathology that have advanced our understanding of neuropsychiatric diseases [5]. Thus far, genetic studies on vulnerable phenotypes, such as posttraumatic stress disorder and major depressive disorder (MDD), have also provided some insights into the genetic factors contributing to resilience [6]. However, these studies have largely focused on outcomes and employed different scales to measure resilience. Here, we set to investigate the underlying genetic component of trait resilience in six cohorts from Germany that measured resilience using a unified scale.

## Methods

### Study sample

In total, six cohorts from Germany contributed to this study, resulting in a collective sample of 15822 adult individuals. A basic description of the study sample composition can be found in Table 1. A description of each independent study is provided in the Supplementary Methods. Briefly, this study included participants with available genotype data and the relevant phenotypic information from the BiDirect Study (N = 1453) [7], the FOR2107 consortium (N = 1789) [8], the PROCAM-2 Study (N = 3879) [9, 10], both SHIP cohorts (SHIP-START/-LEGEND, N = 2230; SHIP-TREND, N = 2330) [11, 12], and the LIFE-Adult-Study (N = 4141) [13, 14]. The independent studies recruited participants of European descent from the population living in and around the cities of Münster, Marburg, Greifswald and Leipzig, Germany. Participants in all cohorts provided written informed consent. Methods were carried out in accordance with the Declaration of Helsinki. The independent studies received approval from the ethics committees at the University of Münster and the Westphalian Chamber of Physicians in Münster, North-Rhine-Westphalia (BiDirect, PROCAM-2), the Universities of Marburg and Münster (FOR2107), the University Medicine Greifswald (SHIP), and the Medical Faculty of the University of Leipzig (LIFE-Adult).

**Table 1:** Basic description of the resilience meta-analysis sample composition.

Dataset	N	Cases (n)	Cases (%)	Age range	Age (mean ± SD)	Females	Males	RS-11 (mean ± SD)
BiDirect Study	1453	Depression (526), ACE (243)	52.92	37–73	56 ± 8	688	765	56 ± 12
FOR2107 consortium	1789	MDD (795), BP (133)	51.87	18–65	36 ± 13	1131	658	56 ± 13
PROCAM-2 Study	3879	Depression (421)	10.85	32–88	60 ± 9	1822	2057	61 ± 10
SHIP-LEGEND	2230	MDD (374)	16.77	29–89	56 ± 14	1177	1053	64 ± 9
SHIP-TREND (B1)	752	MDD (126)	16.76	29–89	57 ± 13	427	325	62 ± 12
SHIP-TREND (B2)	1578	MDD (317)	20.09	28–89	57 ± 14	767	811	61 ± 12
LIFE-Adult-Study	4141	Depression (360)	8.69	18–79	64 ± 13	2166	1975	60 ± 11
Total	15822	Depression (2919), BP (133), ACE (243)	20.83	18–89	55	8178	7644	60

*B1* batch 1, *B2* batch 2, *N* sample size, *BP* bipolar disorder, *MDD* major depressive disorder, *ACE* acute coronary event, *SD* standard deviation, *RS-11* 11-item resilience scale.

### Resilience measurement

The level of trait resilience in all cohorts was assessed using the 11-item Resilience Scale (RS-11), a short version of the original 50-item scale developed in 1993 [3, 4, 15]. The RS-11 examines “personal competence” through nine items and “acceptance of self and life” through two items. The total score can range from 11 to 77, with higher scores indicating higher levels of resilience. This instrument has been validated and standardized in the German population, and has already proven useful to demonstrate the contributions of age, sex, education, socio-economic status, life-satisfaction, self-esteem and social support to resilience [3].

### Genotyping, quality control and imputation

Seven genotype datasets were processed independently by different analysts. Detailed procedures are provided in the Supplementary Methods. Briefly, genome-wide genotyping was performed using different SNP arrays. Genotype calling was conducted as recommended by the array manufacturers in all instances. All genotype datasets underwent common basic quality control (QC) steps, including exclusion of rare variants, variants in Hardy-Weinberg disequilibrium, and variants with low call rates; exclusion of individuals with low genotyping rate, low heterozygosity, high relatedness, sex mismatch, duplicates and outliers. With exception of the PROCAM-2 dataset, which was subjected to a custom pipeline (see Supplementary Methods), datasets were imputed using the 1000 Genomes Project, phase 3 v5 or the Haplotype Reference Consortium (HRC) reference panels using the common SHAPEIT + IMPUTE2 or the standard Michigan Imputation Server [16–18] pipelines. Post-imputation variant filtering to exclude poorly imputed variants (according to Rsq/INFO value) was performed and datasets were further subjected to a second round of QC to exclude imputed variants with very low minor allele frequencies (MAF < 0.01) and in Hardy-Weinberg disequilibrium (HWE p < 1 × 10^−6^).

### Genome-wide association analyses

Independent association analyses were conducted in each dataset applying a common analytic plan. Linear regression was performed with an additive model in Plink 2.0 [19], testing for variant associations with the rank-normalized RS-11 total scores. Because the BiDirect Study and the FOR2107 consortium focus on depression and (to a lesser extent) cardiovascular disease, and on MDD and bipolar disorder, respectively, these cohorts are enriched in disease cases (Table 1). Therefore, all regression models were adjusted for diagnosis in addition to age, sex, education and the first *n* genetic principal components (the appropriate number was selected for each dataset by an experienced analyst). Details can be found in Supplementary Methods.

### Variant- and gene-based meta-analyses

Summary statistics from the seven GWASs were harmonized and a variant-level meta-analysis was performed using the weighted-z method applied in Plink 1.9. From 10093180 variants included in the meta-analysis, 7508201 remained after exclusion of highly heterogeneous variants (i.e. I^2^ heterogeneity index > 40 and p-value for Cochran’s Q statistic < 0.1). Statistical significance was defined using the commonly accepted GWAS threshold for genome-wide associations (p < 5 × 10^−8^). Suggestive associations at the variant-level were defined under the threshold p < 1 × 10^−5^. For the gene-based meta-analysis, all GWAS summary statistics were subjected to gene analysis using MAGMA [20]. Gene boundaries were defined as the start and end positions ±5 kb, according to the Ensembl’s hg19 genome build. The resulting p-values for 20157 markers from these analyses were then meta-analyzed using the fixed-effects with sample size weights method and marker heterogeneity among samples was calculated. Genes that were highly heterogeneous and/or present in less than 60% of the total meta-analysis sample (i.e. with I^2^ > 40 and/or weight < 11113) were excluded. Gene-level genome-wide significance was defined following Bonferroni correction at p < 2.48 × 10^−6^ (0.05/20157). Suggestive candidate genes were defined at p < 1 × 10^−4^.

### Definition and annotation of resilience loci

Independent genomic loci within our variant-based meta-analysis were defined at the suggestive GWAS threshold using the SNP2GENE tool of the Functional Mapping and Annotation of Genome-Wide Association Studies (FUMA-GWAS) platform [21]. Only variants that were present in at least three of the seven datasets were considered for this analysis. Linkage disequilibrium (LD) blocks were formed from variants with p < 0.05 at r^2^ ≥ 0.6 within a 500 kb window according to the 1000 Genomes Project phase 3 European population reference panel. The resulting loci containing only one variant were excluded due to lack of evidence in support of the signal. The SNP2GENE tool was also used to annotate SNPs in the identified loci: 1) genes were mapped to variants according to their start and end positions ±1 kb in the Ensembl’s hg19 genome build; 2) mapping of expression quantitative trait loci (eQTLs) was performed for the variant-gene pairs with false discovery rate (FDR) < 0.05 in the eQTL Catalog, PsychENCODE eQTLs, DICE, BRAINEAC and blood and brain GTEx v8 collections. Details and links to these data sources are available in the tutorial pages of the FUMA-GWAS website (https://fuma.ctglab.nl/tutorial#snp2gene).

### SNP-Heritability (h^2^_SNP_)

The proportion of variance in resilience explained by our variant-level RS-11 meta-analysis was calculated using the LDSC software [22] and the pre-computed LD scores from the 1000 Genomes Project Reference Panel as suggested by the authors.

### Exploration of functional implications

Protein-protein interaction (PPI) data for the genes implicated by the variant- and gene-level meta-analyses at the respective suggestive thresholds was retrieved using the ReactomeFIViz app [23] for Cytoscape 3.9.1 [24]. This analysis used as input 13 genes mapped by chromosomal position and/or eQTL evidence to the suggestive resilience loci from the variant-based meta-analysis and 33 candidates from the gene-based meta-analysis. The resulting PPI network incorporated “linker” genes (i.e. genes not in the input gene list that create indirect connections between input genes) to increase biological interpretability through an analysis of pathway overrepresentation of the PPI data. Overrepresented pathways were considered those that: 1) had FDR < 0.05, 2) did not correspond to specific diseases, such as a type of cancer or infection, 3) had at least three genes overlapping the pathway set, and 4) the overlap with the pathway set represented at least 3% of genes in the set.

### Polygenic scoring

A set of 12 published polygenic scores (PGSs) available at the PGS Catalog [25] were used to approximate the following personality and mental health traits in PROCAM-2: depression, neuroticism, chronotype, self-injurious behavior, educational attainment, general happiness (Publication ID: PGP000263) [26], sensitivity / hurt feelings, suffer from „nerves“, feelings of worry or anxiety, loneliness, friendship satisfaction and health satisfaction (Publication ID: PGP000244) [27] (Table 2). These PGSs were created and evaluated in large samples of predominantly European ancestry and, therefore, are suitable for application to our sample. After downloading and harmonizing weight files, we performed allelic scoring in PROCAM-2 using the sum method applied in Plink 1.9. The association between the rank-normalized PGSs and RS-11 scores was tested through partial correlation tests that used the Pearson model and were adjusted for age, sex, occupation and depression status. Significance was set to FDR < 0.05.

**Table 2:** Correlation between polygenic scores for personality characteristics and RS-11 scores in PROCAM-2.

PGS Catalog ID	Phenotype	Method	Publication ID	# PGS SNPs	# Valid SNPs	% Valid SNPs	r	p	FDR
PGS001829	Depression	Penalized regression (bigstatsr)	PGP000263	7534	7530	99.9	0.0097	0.5453	0.5907
PGS002213	Neuroticism	LDpred2 (bigsnpr)	PGP000263	950183	944901	99.4	−0.0670	3.0 × 10^−5^	0.0004
PGS002209	Chronotype	LDpred2 (bigsnpr)	PGP000263	955439	950000	99.4	−0.0312	0.0522	0.1131
PGS002222	Self-injurious behavior	LDpred2 (bigsnpr)	PGP000263	761279	756897	99.4	−0.0170	0.2912	0.4731
PGS002231	Educational attaintment	LDpred2 (bigsnpr)	PGP000263	950845	945206	99.4	−0.0333	0.0384	0.1131
PGS002154	General happinness	LDpred2 (bigsnpr)	PGP000263	806011	801523	99.4	−0.0505	0.0017	0.0108
PGS001016	Sensitivity / hurt feelings	snpnet	PGP000244	7922	7370	93.0	−0.0329	0.0405	0.1131
PGS001017	Suffer from ‘nerves'	snpnet	PGP000244	3348	3074	91.8	−0.0224	0.1634	0.3034
PGS001021	Feelings of worry or anxiety	snpnet	PGP000244	9747	9110	93.5	−0.0320	0.0464	0.1131
PGS001091	Loneliness	snpnet	PGP000244	660	574	87.0	−0.0136	0.3965	0.5154
PGS001398	Friendship satisfaction	snpnet	PGP000244	659	590	89.5	0.0042	0.7926	0.7926
PGS001401	Health satisfaction	snpnet	PGP000244	1814	1662	91.6	0.0110	0.4920	0.5814

*PGS* polygenic score, *SNPs* single-nucleotide polymorphisms, *FDR* false discovery rate.

## Results

The GWAS meta-analysis of resilience was performed on 10093180 variants and 15822 adult individuals from six German cohorts (Table 1). From the total study sample, 8178 were females (51.7%) and 7644 were males between the ages 18 and 89 years (mean age: 55 years). Collectively, the mean RS-11 score was 60. Notably, we observed that BiDirect and FOR2107 participants had, on average, an RS-11 that was slightly lower (mean: 56 ± 13) than that found in the other cohorts (mean: 61). This reflecting the enrichment of BiDirect and FOR2107 samples in younger participants with a diagnosis of mood or cardiovascular disorders. Overall, depression and bipolar disorder were documented in 18.45% and 0.8% of individuals, respectively.

### Meta-analyses suggested candidate genes for resilience

**Fig. 1:**
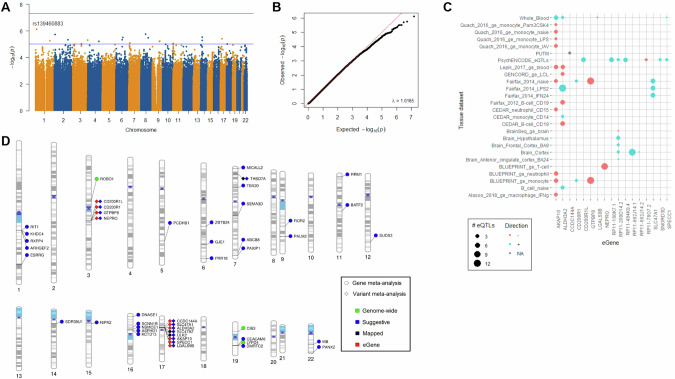
GWAS meta-analyses. **A** Manhattan plot of the variant-based GWAS meta-analysis for resilience (RS-11). No genome-wide signals were found. The red and blue lines depict the genome-wide (p < 5×10^−8^) and suggestive (p < 1×10^−5^) thresholds for statistical significance, respectively. **B** Quantile-quantile plot of the variant-based GWAS meta-analysis. **C** Summary of annotated eQTL effects in brain, blood and immune datasets for variants in suggestive loci. The complete list of eQTL annotations is available in the Suppl. Table 2. **D** Chromosomal location of all potential candidate genes for resilience suggested by variant- and gene-based resilience meta-analyses.

After filtering for heterogeneity, 7508201 variants remained in the summary statistics of the resilience GWAS meta-analysis. No genome-wide signals were found (Fig. 1A, B). The top variant was rs139460883 in chr1:14426083 (p = 7.7 × 10^−7^, Z-score = 4.9). LDSC analysis estimated heritability in the observed scale to be about 6% (h^2^_SNP_ = 0.0594), with lambda GC = 1.0165 and Chi^2^ statistic = 1.0213 indicating validity of this analysis. The distinction of resilience loci using FUMA-GWAS included 6688300 variants present in at least three datasets and identified 11 genomic loci at the suggestive threshold (p < 1 × 10^−5^; Suppl. Table 1). However, we excluded from further analyses six of these that represented single variants, including the top variant, rs139460883. The remaining five suggestive loci formed by at least two variants contained a total of 36 variants, from which 67.6% were located in intergenic regions (Suppl. Figs. S1–S5). Three loci collectively implicated 13 protein-coding genes by physical proximity and/or eQTL annotation (Table 3). Nevertheless, two loci could not be assigned a gene by either mapping approach. Annotation of eQTLs from brain, blood and immune datasets yielded 170 variant-gene-tissue combinations (Fig. 1C; Suppl. Table 2) concerning 27 variants (most frequent eQTL was rs112155453, lead variant of locus #5), 17 genes (most frequent eGene was *ALDH3A2*, near locus #5) and 26 tissues (most frequent tissues were monocytes and collective brain tissues from PsychENCODE).

**Table 3:** Genomic loci suggested for trait resilience through variant-based meta-analysis.

Locus	Lead variant	Lead rsID	Lead p	Size (bp)	Start (bp)	End (bp)	# SNPs	Protein-coding Mapped/eQTL Genes
1	3:112515187:A:G	rs6797028	9.4 × 10^−6^	5812	112512647	112518459	12	*CD200R1L, CD200R1, GTPBP8, NEPRO*
2	3:193598924:C:T	rs78180970	4.9 × 10^−6^	19899	193598924	193618823	3	*–*
3	7:11555542:C:G	rs17633522	8.1 × 10^−6^	7089	11554555	11561644	4	*THSD7A*
4	14:40818021:C:T	rs61989120	3.0 × 10^−^^6^	96875	40763557	40860432	12	*–*
5	17:19771057:C:G	rs112155453	3.9 × 10^−^^6^	164542	19612489	19777031	5	*CCDC144A, SLC47A1, ALDH3A2, SLC47A2, ULK2, AKAP10, SPECC1, LGALS9B*

*bp* base pairs, *SNPs* single-nucleotide polymorphisms, *eQTL* expression quantitative trait loci.

The gene-based meta-analysis included 20157 genes present in the summary statistics of our datasets. At the Bonferroni-corrected genome-wide threshold of significance (p < 2.48 × 10^−6^), we found three genes associated with resilience (Table 4), namely *ROBO1* (Roundabout Guidance Receptor 1), *CIB3* (Calcium And Integrin Binding Family Member 3) and *LYPD4* (LY6/PLAUR Domain Containing 4). Moreover, at the explorative threshold (p < 1 × 10^−4^), 30 protein-coding genes were suggested as potential candidate genes for resilience (Table 4).

**Table 4:** Protein-coding candidate genes suggested for trait resilience through gene-based meta-analysis.

Symbol	Chr	Start	End	Weight	Z	p	I^2^	Het.p
*RIT1*	1	155867599	155881195	15799	4.402	1.1 × 10^−5^	0	0.748
*KHDC4*	1	155882834	155904191	15798	4.354	1.3 × 10^−^^5^	0	0.737
*RXFP4*	1	155911480	155912625	15791	4.370	1.2 × 10^−^^5^	0	0.770
*ARHGEF2*	1	155916630	155976861	15793	4.328	1.5 × 10^−^^5^	0	0.774
*ESRRG*	1	216676588	217311097	15793	3.906	9.4 × 10^−5^	0	0.996
***ROBO1***	**3**	**78646390**	**79816965**	**15794**	**5.029**	**4.9** × **10**^**−7**^	**0**	**0.991**
*PCDHB1*	5	140430979	140433512	15795	3.948	7.9 × 10^−5^	12.5	0.335
*ZBTB24*	6	109783797	109804440	15799	3.935	8.3 × 10^−5^	0	0.883
*GJE1*	6	142454227	142456288	15801	4.391	1.1 × 10^−5^	6.5	0.378
*PRR18*	6	166719168	166721936	15789	3.952	7.7 × 10^−5^	0	0.706
*MICALL2*	7	1468101	1499138	11643	3.971	7.2 × 10^−5^	0	0.717
*TBX20*	7	35242042	35293758	15795	3.913	9.1 × 10^−5^	0	0.821
*SEMA3D*	7	84624869	84816171	15793	4.078	4.5 × 10^−5^	11.6	0.341
*ABCB8*	7	150725510	150744869	11654	4.457	8.3 × 10^−^^6^	0	0.998
*PAXIP1*	7	154735397	154794794	15799	4.398	1.1 × 10^−5^	0	0.734
*ROR2*	9	94325373	94712444	15797	4.313	1.6 × 10^−5^	0	0.570
*PALM2*	9	112403068	112713755	15795	3.988	6.7 × 10^−5^	0	0.639
*RRM1*	11	4115937	4160106	15795	4.209	2.6 × 10^−5^	33.4	0.173
*BATF2*	11	64755415	64764517	15795	4.248	2.2 × 10^−5^	0	0.664
*SUDS3*	12	118814185	118855840	15800	4.083	4.5 × 10^−5^	0	0.988
*SDR39U1*	14	24908972	24912111	15802	3.893	9.9 × 10^−5^	0	0.514
*NIPA2*	15	23004684	23034427	15787	3.958	7.6 × 10^−5^	39.7	0.127
*DNASE1*	16	3661729	3730144	15789	3.972	7.1 × 10^−5^	0	0.658
*SCNN1B*	16	23289552	23392620	15794	4.005	6.2 × 10^−5^	0	0.434
*NSMCE1*	16	27236312	27280115	15793	4.014	6.0 × 10^−5^	0	0.529
*ASPHD1*	16	29911696	29931185	15786	3.981	6.9 × 10^−5^	0	0.813
*KCTD13*	16	29916333	29938356	15783	4.033	5.5 × 10^−5^	0	0.799
***CIB3***	**19**	**16272179**	**16284336**	**15790**	**4.752**	**2.0** × **10**^**−6**^	**0**	**0.691**
*CEACAM6*	19	42254885	42276113	15791	4.246	2.2 × 10^−5^	0	0.832
***LYPD4***	**19**	**42341148**	**42348760**	**15786**	**4.778**	**1.8** × **10**^**−6**^	**0.3**	**0.421**
*DMRTC2*	19	42348806	42356401	15793	4.031	5.6 × 10^−5^	29.8	0.201
*MB*	22	36002811	36033998	15788	4.563	5.0 × 10^−6^	22.2	0.260
*PANX2*	22	50609160	50618723	15782	4.176	3.0 × 10^−5^	0	0.676

Genome-wide significant (p < 2.48×10^−6^) genes are highlighted in bold.

**Fig. 2:**
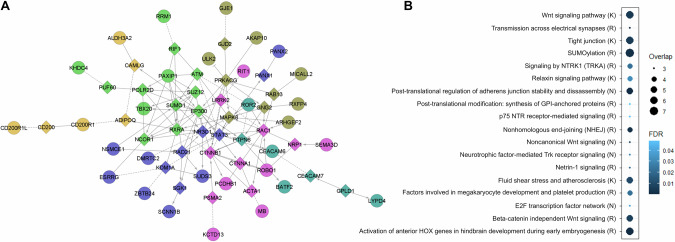
Biological context. **A** Protein-protein interaction network. Input genes were those depicted in Fig. 1C. Linker genes different from the resilience suggested candidates were added to increase network connectivity and interpretability. Successfully integrated input genes are shown in circles. Linker genes are shown in diamonds. Colors represent clusters of genes with high functional interaction scores. Arrows indicate the direction of the interaction, whereas dotted lines indicate predicted interactions. **B** Network pathways fulfilling criteria for overrepresentation that overlapped input genes. The complete list of overrepresented pathways in the network is available in the Suppl. Table 4. K: KEGG pathway, R: Reactome pathway, N: NCI-Nature pathway.

To enable a systematic biological interpretation of these suggestive findings, we leveraged PPI information. We used all 46 protein-coding genes identified by both meta-analysis approaches (Fig. 1D; Suppl. Table 3) as input to build a network that incorporated linker genes. This was later analyzed for overrepresented pathway gene sets. The analysis resulted in a network containing 30 of the resilience input genes and incorporated other 33 genes as linkers, including *EP300* (E1A Binding Protein P300) as hub node (Fig. 2A). The pathway analysis showed an overrepresentation of biological processes involved in neuronal development and function (e.g. proliferation, differentiation, migration, synaptic organization), immunity and vascular homeostasis (Suppl. Table 4). Overrepresented pathways that overlapped network input genes are shown in Fig. 2B.

### Polygenic scores for two mental health traits correlated with trait resilience

Finally, to test whether personality and mental health traits are linked with the levels of resilience at the genetic level, 12 publicly available PGSs were calculated in PROCAM-2 (Table 2). There was a good overlap between PGS and PROCAM-2 variants in all instances (87-99.9%), validating the utility of these instruments in our study. Partial correlation analyses with RS-11 revealed inverse relationships between the levels of resilience and the PGSs for “neuroticism” and “general happiness”. Here, however, as the categories for “general happiness” are inversely coded (UK Biobank Data-Field 20458, Data-Coding 537; 1 = extremely happy, through 6 = extremely unhappy), our finding indicates that the genetic determinants of higher general happiness correlate with higher levels of resilience, hence denoting a positive relationship with this trait. Of note, as observed in Table 2, the PGSs for intelligence, sensitivity and anxiety proxy phenotypes, namely educational attainment, “hurt feelings” and “feelings of worry or anxiety”, respectively, also showed nominal significance (p < 0.05) and a negative relationship with RS-11. However, the latter findings did not survive correction for multiple comparisons.

## Discussion

To uncover genetic factors that contribute to trait resilience, we combined variant- and gene-based GWAS meta-analyses from six German cohorts (N = 15822) using as outcome measure the RS-11 scores, and investigated the biological context using a network approach. Moreover, we explored the relationship between resilience and the genetic determinants of other personality and mental health traits using PGSs. These analyses found three genes (*ROBO1*, *CIB3* and *LYPD4*) associated with resilience and suggested another 53 potential candidate genes (protein-coding + non-coding genes; Suppl. Table 3). The identified genes participate in processes important for brain development, immunity and vascular homeostasis. In addition, we observed a relationship between resilience and the genetic determinants of personality and mental health traits, in particular neuroticism and general happiness.

Previously, a GWAS of resilience conducted in about 11500 U.S. Army soldiers participating in the Army Study To Assess Risk and Resilience in Servicemembers (STARRS) reported the association of a small intergenic locus in chromosome 4, near *DCLK2*, and of the gene *KLHL36* with resilience [28]. Although we did not find association signals in those genes previously reported by Stein et al. in a sample of similar ancestry and size, it should be noted that there were core differences between our studies. Perhaps most importantly, Stein et al. used a 5-item self-report questionnaire to measure psychological resilience in a highly specific population, while our study was aimed at a more general adult population, and the comparability of resilience measurements between this 5-item self-report questionnaire and the RS-11, to our knowledge, has not been determined. Nevertheless, because *DCLK2* is crucial for proper hippocampal organization and function [29], the study also indicated that brain development may play a central role in the establishment of resilience. Moreover, considering both studies relatively comparable in size and design within their own contexts, the low rate of genome-wide significant findings in both suggests a low heritability of trait resilience. This, however, contradicts the large heritability estimated from twin studies (up to 52%) [30–33]. Likely, large international collaborations will be required in future studies to reach sample sizes with sufficient statistical power to clarify these contradictory results and successfully uncover the genetic factors contributing to trait resilience in the general population. However, the comparability between instruments that measure resilience should be assessed first, given that the heterogeneity of conceptualizations and measurements has been shown to lead to inconsistency in the results and difficulty in comparing studies [34–36].

In our study, network analysis suggested the involvement of various pathways related to brain development, including Wnt, Notch, Rac1, thyroid hormone (TH) and neurotrophin signaling, as well as to immune and stress response pathways, such as B cell receptor and glucocorticoid receptor signaling, and vascular homeostatic processes, including fluid shear stress and cadherin signaling, in trait resilience. Such pathways also overlap and form complex interactions that influence mental health. For example, the Wnt pathway has been shown to indirectly regulate TH function and has been tied to thyroid development and homeostasis as well as to the expression of TH receptors and deiodinases (D1-D3) in TH target tissues. At the same time, THs regulate tissue development and homeostasis [37]. In the brain, THs are not only essential for proper development and function through the lifespan, but they also influence mood and behavior. Therefore, thyroid dysfunction is a known risk factor for psychiatric conditions, including depressive, bipolar and anxiety disorders [38]. Importantly, the immune system is crucial for brain development, participating in cell survival, proliferation, migration and differentiation, axonal growth, synaptogenesis, synaptic remodeling and dendritic pruning. Moreover, chemokines and toll-like receptors are known regulators of cognitive function and behavior [39]. In addition, neurovascular function can be influenced by inflammatory signaling, and compromise of the blood-brain-barrier has been previously observed in the context of vulnerability and resilience to stress [40, 41]. These findings are in agreement with previous observations coming from neurobiological and molecular studies of resilience, which have shown involvement of various neurotransmitter systems, hormones and neuropeptides in resilience, as well as alterations in neural circuits regulating emotion and social behavior, among others [42].

The involvement of such processes was, however, not only observed through network pathway analysis, which might have been biased due to the inclusion of linker genes, but was also supported when querying the genes identified through both meta-analysis approaches in the GeneCards Human Gene Database (https://www.genecards.org/; accessed in June 2023). For example, from the variant-based analysis, summaries of the functions of *NEPRO* and *ULK2* place them as participants of cortex development and maintenance of neural progenitors [43], and of neuronal differentiation [44], respectively, while those of *CD200R1L* and *CD200R1* suggest these function as inhibitors of inflammation [45]. The GWAS Catalog (https://www.ebi.ac.uk/gwas/; accessed in June 2023) also offered important insights into previously reported genetic associations with relevant traits. Here, for example, *NEPRO* and *ULK2* are associated with psychotic symptoms in Alzheimer’s disease [46] and with cortical thickness [47], respectively, while *CD200R1L* and *CD200R1* show associations with various immune traits, such as the proportion of eosinophils and neutrophils in blood, levels of cortisol, and inflammatory diseases like Crohn’s disease and rheumatoid arthritis. Moreover, rs1952935, which supports the signal of the top locus (#4), has a reported association with risk-taking behavior [48].

Similar was the case for the suggestive results from the gene-based analysis, where GeneCards queries led to the identification of processes participating in brain development and synaptic function being represented by genes such as *RIT1, ARHGEF2*, *PCDHB1*, *MICALL2*, *SEMA3D*, *KCTD13* and *PANX2*, while processes related to immune activity were represented by genes such as *BATF2*, *PAXIP1*, *DNASE1* and *CEACAM6*. In the GWAS Catalog, some genes appeared to be of particular interest, including *ESRRG*, with reported associations with cognitive performance and executive function, risk-taking and externalizing behaviors, anhedonia, major depression and other mental health-related traits; *TBX20*, associated with cardiovascular disease and suicidal behavior; *PAXIP1*, associated with cognitive performance, intelligence and brain volume; *PALM2*, which was associated with various traits related to cognition, vascular function and number of immune cells; and *SUDS3*, associated with loneliness, neuroticism, educational attainment and depression. Because an extensive discussion of each of the suggestive findings from our study is beyond the aim of our report, we would like to refer the reader to the source databases (i.e. GeneCards and GWAS Catalog) for more details and links to the respective publications. For the purposes of this discussion, all of the above seems to convey evidence indicating that the genes and loci identified in our study participate in developmental and immune processes that have previously shown to also impact mental health traits. In very general terms, these observations are in agreement with those genetic associations reported for resilience when considered as a process, which include genes with important functions in development and the inflammatory and stress responses, such as *BDNF*, *COMT*, *NPY*, *IL6* and *IL10* [35].

The gene-based meta-analysis of resilience found three genome-wide significant signals, corresponding to *ROBO1*, *CIB3* and *LYPD4*. Although the function of *LYPD4* is unknown, this gene appears to be associated with serum levels of protein PCDHGA1 [49], which may be involved in the establishment and maintenance of specific neuronal connections in the brain [50]. *ROBO1* functions in axon guidance, neuronal precursor cell migration and interaural interaction in auditory pathways [51–53]. The gene has been associated with various mental health-relevant traits, including cognitive function measurement, information processing speed, unipolar depression, depressive symptoms, facial emotion recognition, schizophrenia, cortical thickness and other brain measurements, educational attainment, mathematical ability and blood pressure (source: GWAS Catalog). Interestingly, *CIB3* encodes an auxiliary subunit of the sensory mechanoelectrical transduction (MET) channel in cochlear hair cells [54], which places a second resilience-associated gene in the auditory system. Sensory processing difficulties in mental disorders other than autism spectrum disorders are largely understudied. However, there is evidence that individuals with depression, bipolar disorder and schizophrenia, among other mental health problems, show patterns of sensory processing that differ from those in healthy individuals [55]. In particular, some studies have also proposed neuroanatomical correlates of (stress) resilience that involve the auditory system, including activity of the amygdala and a thalamic-primary auditory cortex circuit [56, 57]. Therefore, if we consider resilience as both a trait and a dynamic process, as suggested by Fares-Otero et al. [58], this finding opens the possibility for the implementation of interventions, such as music therapy, to promote resilience for the prevention and treatment of mental health problems.

We acknowledge that relying on the RS-11 measure of resilience importantly limited our ability to consider more cohorts for inclusion in our meta-analysis, resulting in a relatively small sample size that prevented the identification of genome-wide associations at the variant-level. However, reviews of the resilience literature have repeatedly emphasized the need for consistency among studies to advance research in the field [34–36]. With this in mind, we favored homogeneity over increasing size in our study. The inclusion of only cohorts from the German population is another limitation of our study. The applicability of our findings to individuals from other nationalities and ancestries remains to be investigated. Therefore, efforts to collect resilience measurements using unified instruments in large international cohorts to unravel the genetics of trait resilience in the general population should be encouraged. This would also enable the investigation of the genetic correlation between resilience and personality and mental health traits, which was not possible in our study due to the lack of full summary statistics for the personality and mental health traits, and that of independent samples suitable for the generation of a resilience PGS derived from our GWAS meta-analysis. Despite these limitations, our study represents, to our knowledge, the largest investigation of the genetics of trait resilience to date, and provides initial and valuable insights into the heritability and biology of resilience in the general population, its relationship with the genetics of personality traits and mental health, and future directions in the field. Understanding the biological basis of trait resilience holds the potential to aid in the development of preventive strategies for mental health conditions through the promotion of higher levels of resilience, particularly in at-risk individuals.

## Supplementary information

Supplementary Methods

Supplementary Figures

Supplementary Tables

## Data Availability

All data supporting the findings of this study are available within the paper and its Supplementary Information. Full summary statistics are available from the corresponding author upon reasonable request.
