# Supplementary material for: A meta-analysis of genome-wide studies of resilience in the German population
Source: Mol Psychiatry. 2024 Aug 8;30(2):497–505. doi: 10.1038/s41380-024-02688-1 (PMC11746137; doi:10.1038/s41380-024-02688-1)
Supplement: Supplementary file 1 — Supplementary Methods [file 41380_2024_2688_MOESM1_ESM.pdf]

A meta-analysis of genome-wide studies of resilience in the German population

SUPPLEMENTARY METHODS

1. Independent Cohorts (study population)

BiDirect Study. The BiDirect Study is a prospective, observational study to explore the bidirectional relationship between depression and subclinical arteriosclerosis. The sample is comprised of community-dwelling adults (control cohort), patients with an acute depressive episode, and patients who recently suffered from an acute coronary event (ACE). All study participants were recruited in the district of Münster, Germany, and underwent extensive phenotyping during a 10-year period with three follow-ups. The study design and methods have been previously described in detail [Teismann H et al., 2014]. The first assessment of resilience was conducted during the first follow-up in 2013-2016. All participants of the BiDirect Study provided written informed consent. Methods were carried out in accordance with the ethical standards laid down in the updated version of the 1964 Declaration of Helsinki. The BiDirect Study was approved by the ethics committee of the University of Münster and the Westphalian Chamber of Physicians in Münster, North-Rhine-Westphalia, Germany.

FOR2107. The FOR2107 consortium was established to investigate the neurobiology of affective disorders. The clinical and neurobiological effects of genetic and environmental factors on disease etiology and progression are studied using animal models and humans. The cohort consists of 2,500 participants in the following groups: patients with affective disorders, such as major depression and bipolar disorder, healthy individuals at high risk of

developing these, and a group of control individuals. Participants were recruited in and around the cities of Marburg and Münster, Germany, and underwent extensive phenotyping at baseline and follow-up. The study design and methods have been described elsewhere [Kircher T et al., 2019]. The assessment of resilience was conducted in 2014-2018. The study protocols were approved by the ethics committees of the Medical Schools of the Universities of Marburg and Münster, following the Declaration of Helsinki. All participants provided written informed consent.

PROCAM-2 Study. The Prospective Cardiovascular Münster (PROCAM) Study is a large, prospective epidemiological study of cardiovascular risk factors and diseases performed in an occupational cohort. Detailed study descriptions can be found elsewhere [Assmann G et al., 2002; Voss R et al., 2002]. For this analysis, we used data of PROCAM-2, the study's second part in which participants were examined between 2000 and 2007. For PROCAM-2, 17,312 employees were recruited in more than 50 regional companies and government authorities on a voluntary basis. All underwent a cardiovascular examination at baseline. Follow-up assessments ceased until 2018, when the cohort was „revived“ for genotyping and a follow-up investigation was performed via mailed questionnaires in 2021. This included the assessment of resilience. The study was conducted in accordance with the Declaration of Helsinki and was approved by the ethics committee of the University of Münster and the Westphalian Chamber of Physicians in Münster, North-Rhine-Westphalia, Germany .

SHIP cohorts. The Study of Health in Pomerania (SHIP) is a population-based project to investigate disease incidences and the relationships between common risk factors, subclinical disorders and disease outcomes on a longitudinal scale. It consists of two independent cohorts: SHIP-START and SHIP-TREND. These cohorts have been described in detail elsewhere [Völzke H et al., 2011, 2022]. Briefly, the study population for SHIP-START was sampled from the general adult population (20-79 years old) of West Pomerania, Northeast Germany. At baseline (recruited between 1997 and 2001) the cohort

comprised 4,308 participants who underwent extensive phenotyping and various follow-up assessments. A special follow-up of 2,400 participants, referred to as SHIP-LEGEND, with in-depth diagnostic interviews and assessments for personality factors, including resilience, and mental disorders was conducted between 2007 and 2010. The study population for SHIP-TREND was sampled similarly to SHIP-START. At baseline (recruited between 2008 and 2012) the cohort comprised 4,420 participants. The first follow-up was conducted between 2016 and 2019 in 2,507 participants and included the assessment of resilience. The investigations in SHIP were carried out in accordance with the Declaration of Helsinki, including written informed consent from all participants. The survey and study methods were approved by the Ethics Committee at the University Medicine Greifswald, Germany.

LIFE-Adult-Study. The Leipzig Research Centre for Civilization Diseases (LIFE) Adult Study is a population-based cohort study conducted in the city of Leipzig, Germany. It was initiated in 2009 with the purpose of investigating various aspects of major diseases of public health concern, including prevalence, early detection, and the role of genetics and life style in cardiovascular and neurocognitive disorders. Participants were recruited in 2011-2014. The cohort consists of 10,000 Leipzig residents aged 18-79 years that underwent an extensive core assessment programme at baseline. Details of the study are published elsewhere [Loeffler M et al., 2015; Engel C et al., 2022]. The follow-up assessment in which resilience was assessed for the first time was conducted in 2017-2021. All procedures were carried out in accordance with the Declaration of Helsinki. Written informed consent was obtained from all participants. The study was approved by the institutional ethics board of the Medical Faculty of the University of Leipzig.

2. Sampling and Genotyping

BiDirect Study. Genomic DNA was isolated from whole blood samples with EDTA using standard DNA extraction kits and procedures at the University of Münster. Genome-wide genotyping was performed with the Infinium PsychArray BeadChip v1 (Illumina) at Life&Brain GmbH (Bonn, Germany). Basic quality control (QC) was employed to remove samples and variants with high rates of missing data. This included removal of individuals with genotyping rate <98%, cryptic relatedness (PI-HAT ≥1/16), sex mismatch and genetic outliers (distance in first two multidimensional scaling components >5 standard deviations from the mean), as well as the removal of variants with call rate <98% and minor allele frequency (MAF) <1%. Genotype imputation to the HRC v1.1 reference panel was performed using the pipeline implemented in the Michigan Imputation Server (phasing with Eagle2 and imputation with Minimac4 software). Post-imputation QC was performed with the plinkQC R package on variants filtered according to INFO metric (≥0.8). The final dataset consisted of 1,453 individuals and 6,800,683 variants.

FOR2107. Genomic DNA was isolated from whole blood samples using standard extraction methods. Genome-wide genotyping was performed with the Infinium PsychArray BeadChip v1 (Illumina). QC steps on samples included removal of individuals with genotyping rate <98%, cryptic relatives (relatedness >12.5%), and genetic population outliers. QC steps on variants included removal of variants with call rate <98% and MAF <1%. Imputation was conducted with SHAPEIT2 (pre-phasing) and IMPUTE2 in 5–megabase pair chunks using the HRC v1.1 reference panel. Imputed variants were filtered for MAF (≥1%), INFO metric (≥0.8), and HWE ($p \geq 1x10^{-6}$). The final dataset consisted of 1,789 individuals and 5,164,663 variants.

PROCAM-2 Study. Genomic DNA was isolated from whole blood samples collected at baseline assessment using standard extraction methods. Genotyping was performed with

the Global Screening Array-24+ vs.1.0 (GSA + Multi-Disease, Illumina) for 9,484 participants. The Genotyping Module v2.0.3 of GenomeStudio v2.0 was used for genotype calling. During the pre-imputation QC, variants with alleles other than A,C,T,G, ambiguous position and/or ID, call rate <98%, MAF ≤5% and HWE p≤1x10^{-5} were excluded. Although no samples were removed prior to imputation, variant QC criteria were applied only for samples with call rate >90%, with non-ambiguous sex calls, non-related (PI-HAT ≤0.09) and that did not represent outliers in a principal components analysis (PCA). Imputation was performed using a custom pipeline that utilized Eagle2 (v2.0.5) and the Positional Burrows Wheeler Transform (pbwt, v3.1), and the UK10K + 1000 Genomes Project, phase 3, reference panels. Imputed variants were filtered for the INFO metric (≥0.8), MAF (≥1%) and HWE (p≥1x10^{-6}). Individuals were removed when a failed heterozigosity test or cryptic relatedness (PI-HAT >0.25) were observed using the plinkQC R package. The final dataset consisted of 3,879 individuals and 8,336,778 variants.

SHIP cohorts. Blood samples were collected at baseline assessment. Non-fasting blood samples were drawn from the cubital vein in the supine position. The samples were taken between 07:00 AM and 04:00 PM.

The SHIP-START samples were genotyped using the Affymetrix Genome-Wide Human SNP Array 6.0. Hybridization of genomic DNA was done in accordance with the manufacturer's standard recommendations. Genotypes were determined using the Birdseed2 clustering algorithm. Arrays with a genotyping call rate <86%, duplicates (by IBS), and mismatches between reported and genotyped sex were removed. Imputation of genotypes was performed using the HRC v1.1 reference panel and the Eagle2 and Minimac3 software implemented in the Michigan Imputation Server for pre-phasing and imputation, respectively. SNPs with HWE p<0.0001, a call rate <95%, or monomorphic SNPs were removed before imputation. Imputed variants were filtered for the INFO metric (≥0.8), MAF (≥1%) and HWE (p≥1x10^{-6}). The final dataset consisted of 2,230 individuals and 7,030,080 variants.

Genotyping of SHIP-TREND samples was performed in two different batches:

The first subset of the SHIP-TREND samples (B1) was genotyped using the Illumina Human Omni 2.5 array. Hybridization of genomic DNA was done in accordance with the manufacturer's standard recommendations at the Helmholtz Zentrum München (Munich, Germany). Genotypes were determined using the GenomeStudio Genotyping Module v1.0 (GenCall algorithm). Arrays with a genotyping call rate <94%, duplicates (based on estimated IBD), and mismatches between reported and genotyped sex were removed. Imputation of genotypes was performed using the HRC v1.1 reference panel and the Eagle2 and Minimac3 software implemented in the Michigan Imputation Server for pre-phasing and imputation, respectively. SNPs with a HWE p<0.0001, call rate <95%, or monomorphic SNPs were removed before imputation. Imputed variants were filtered for the INFO metric (≥0.8), MAF (≥0.01) and HWE ($p \geq 1x10^{-6}$). The final dataset consisted of 752 individuals and 7,418,195 variants.

The second subset of the SHIP-TREND samples (B2) was genotyped using the Illumina GSA chip. Hybridization of genomic DNA was done in accordance with the manufacturer's standard recommendations at Life&Brain GmbH (Bonn, Germany). Genotypes were determined using the GenomeStudio 2.0 Genotyping Module (GenCall algorithm). Prior to quality control, SNPs with a minor allele count ≤10 were excluded. Arrays with a genotyping call rate <94%, duplicates (based on estimated IBD), mismatches between reported and genotyped sex, genetic PCA outliers (>8 SD of the mean in one of the first 10 principal components -PCs- in 5 iterations), and arrays with extreme heterozygosity (>4 SD of the mean) were removed. SNPs with HWE p<0.0001, call rate <95%, and MAF <1% or a minor allele count <10 were removed before imputation. Imputation of genotypes was performed using the HRC v1.1 reference panel and the Eagle and minimac3 software implemented in the Michigan Imputation Server for pre-phasing and imputation, respectively. Imputed

variants were filtered for the INFO metric (≥0.8), MAF (≥1%) and HWE ($p \geq 1x10^{-6}$). The final dataset consisted of 1,578 individuals and 7,306,608 variants.

LIFE-Adult-Study. Blood sampling procedures are detailed elsewhere [Loeffler M et al., 2015]. Participants of LIFE-Adult were genotyped using the Affymetrix Axiom CEU1 micro-array platform. Genotype calling was performed as recommended by the manufacturer using Affymetrix Power Tools v1.20.06. Sample filtering comprised exclusions according to dishQC <0.82, call rate <97%, mismatch of genotyped and reported sex, cryptic relatedness and outliers (6SD of the first 10 PCs). SNP filtering comprised exclusion based on call rate <97%, cluster plot irregularities (assessed by the Affymetrix cluster plot quality metrics), monomorphic SNPs, SNPs violating HWE ($p<1x10^{-6}$) and SNPs showing batch effects ($p<1x10^{-7}$). Genotypes were imputed to the 1000 Genomes Project, phase 3 (v5), using SHAPEIT and IMPUTE2. Imputed variants were filtered for the INFO metric (≥0.8), MAF (≥1%) and HWE ($p \geq 1x10^{-6}$). The final dataset consisted of 4,141 individuals and 8,973,148 variants.

3. Independent Genome-Wide Association Analyses

All GWASs were performed following the same methodology. For each dataset, RS-11 scores were transformed to achieve normal distribution using the rank-based inverse normal transform (INT) applied with the rankNorm function of the RNOmni R package. Association analyses were carried out in Plink 2.0 using linear regression assuming an additive model of inheritance and variance standardization of the covariates used in the model. The models used for each dataset are specified bellow:

BiDirect Study. The model was adjusted for sex, age at RS-11 assessment, schooling years, primary diagnosis (depression or ACE status) and the first 5 genetic PCs.

FOR2107. The model was adjusted for sex, age at RS-11 assessment, schooling years, outcome (depression or bipolar disorder status) and the first 2 genetic PCs.

PROCAM-2 Study. The model was adjusted for sex, age at RS-11 assessment, occupation, depression status and the first 15 genetic PCs. Because information on years of education and/or education level were not available for this cohort, we used the field “occupation“ as a „proxy“. Occupations were classified into 9 categories as follows: 1=civil servant, 2=clerk, 3=student, 4=housewife, 5=worker, 6=craftsman, 7=farmer, 8=self-employed and 9=pensioner.

SHIP cohorts. The GWASs were performed separately but uniformly for SHIP-START, SHIP-TREND B1 and SHIP-TREND B2. Models were adjusted for age (when answering the RS-11 questionnaire), sex, lifetime depression status, schooling years (factorized based on the German school system as 1: <10 years of schooling, 2: 10 years, 3: >10 years) and the first 5 genetic PCs.

LIFE-Adult-Study. The model was adjusted for age, sex, depression status, education (factorized in three categories based on the CASMIN educational classification) and the first 10 genetic PCs.

Data References

Teismann H, Wersching H, Nagel M, et al. Establishing the bidirectional relationship between depression and subclinical arteriosclerosis--rationale, design, and characteristics of the BiDirect Study. *BMC Psychiatry*. 2014;14:174.

Kircher T, Wöhr M, Nenadic I, et al. Neurobiology of the major psychoses: a translational perspective on brain structure and function-the FOR2107 consortium. *Eur Arch Psychiatry Clin Neurosci.* 2019;269(8):949-962.

Assmann G, Cullen P, Schulte H. Simple scoring scheme for calculating the risk of acute coronary events based on the 10-year follow-up of the prospective cardiovascular Münster (PROCAM) study. *Circulation*. 2002;105(3):310-315.

Voss R, Cullen P, Schulte H, Assmann G. Prediction of risk of coronary events in middle-aged men in the Prospective Cardiovascular Münster Study (PROCAM) using neural networks. *Int J Epidemiol*. 2002;31(6):1253-1264.

Völzke H, Alte D, Schmidt CO, et al. Cohort profile: the study of health in Pomerania. *Int J Epidemiol.* 2011;40(2):294-307.

Völzke H, Schössow J, Schmidt CO, et al. Cohort Profile Update: The Study of Health in Pomerania (SHIP). *Int J Epidemiol.* 2022;51(6):e372-e383.

Loeffler M, Engel C, Ahnert P, et al. The LIFE-Adult-Study: objectives and design of a population-based cohort study with 10,000 deeply phenotyped adults in Germany. *BMC Public Health*. 2015;15:691.

Engel C, Wirkner K, Zeynalova S, et al. Cohort Profile: The LIFE-Adult-Study. *Int J Epidemiol.* 2022;dyac114.

Software Links

GenomeStudio (Illumina):
https://support.illumina.com/array/array_software/genomestudio/downloads.html

Analysis Power Tools (Affymetrix):
https://www.affymetrix.com/support/developer/powertools/changelog/index.html

Eagle2: https://alkesgroup.broadinstitute.org/Eagle/

pbwt: https://github.com/richarddurbin/pbwt

SHAPEIT(2): https://mathgen.stats.ox.ac.uk/genetics_software/shapeit/shapeit.html#home

IMPUTE2: https://mathgen.stats.ox.ac.uk/impute/impute_v2.html

Michigan Imputation Server (Eagle2, Minimac3/4):
https://imputationserver.sph.umich.edu/index.html#!

plinkQC (R package): https://cran.r-project.org/web/packages/plinkQC/index.html

RNOmni (R package): https://cran.r-project.org/web/packages/RNOmni/index.html

Plink 2.0: https://www.cog-genomics.org/plink/2.0/
