# Supplementary material for: A meta-analysis of genome-wide studies of resilience in the German population
Source: Mol Psychiatry. 2024 Aug 8;30(2):497–505. doi: 10.1038/s41380-024-02688-1 (PMC11746137; doi:10.1038/s41380-024-02688-1)

A meta-analysis of genome-wide studies of resilience in the German population

SUPPLEMENTARY FIGURES

Suppl.Figure.S1. ***FUMA-GWAS analysis of variant-based meta-analysis: locus #1.*** Suggestive locus localized at chr3:112512647-112518459 (5,812 bps). It encompassed 12 variants with at least nominal significance ($p<0.05$), and implicated six genes by physical proximity and/or eQTL annotation in brain, blood and/or immune cells. The locus was led by the variant rs6797028 ($p=9.4 \times 10^{-6}$, Z= -4.4).

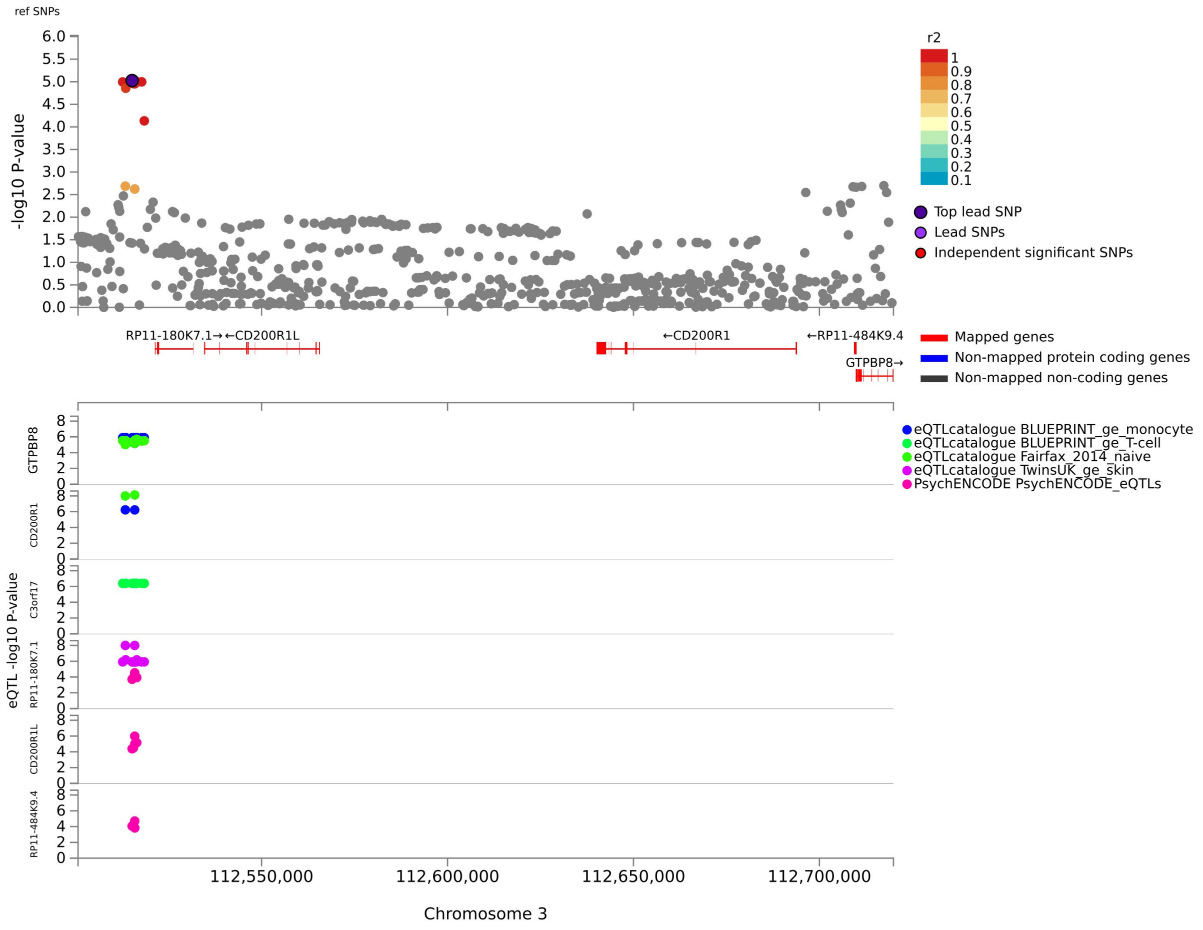

Suppl.Figure.S2. ***FUMA-GWAS analysis of variant-based meta-analysis: locus #2.*** Suggestive locus localized at chr3:193598924-193618823 (19,899 bps). It encompassed three variants with at least nominal significance ($p<0.05$). The locus was led by the variant rs78180970 ($p=4.9\times10^{-6}$, Z= -4.6).

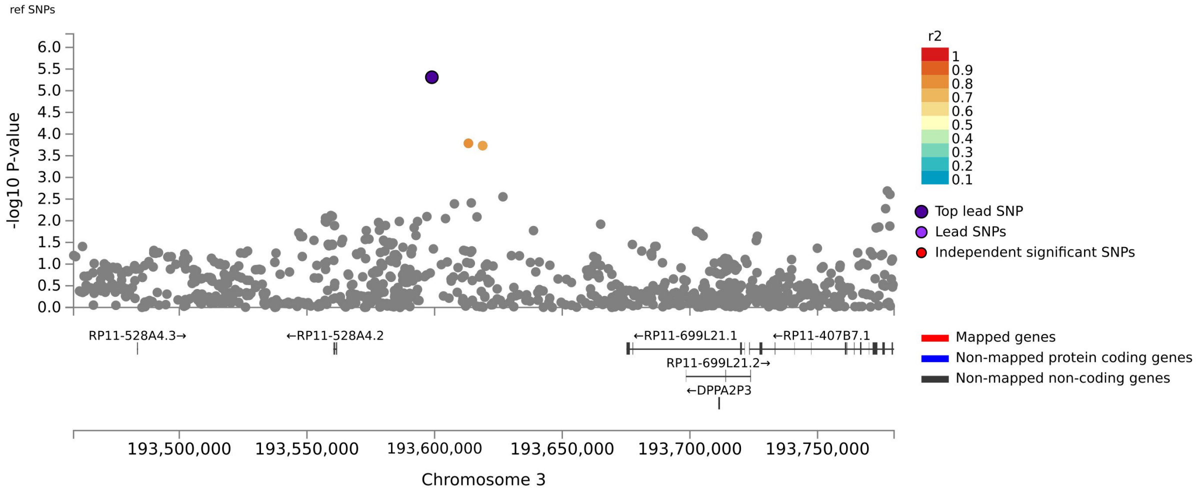

Suppl.Figure.S3. ***FUMA-GWAS analysis of variant-based meta-analysis: locus #3.*** Suggestive locus localized at chr7:11554555-11561644 (7,089 bps). It encompassed four variants with at least nominal significance ($p<0.05$). The locus was led by the variant rs17633522 ($p=8.1\times10^{-6}$, Z= -4.5).

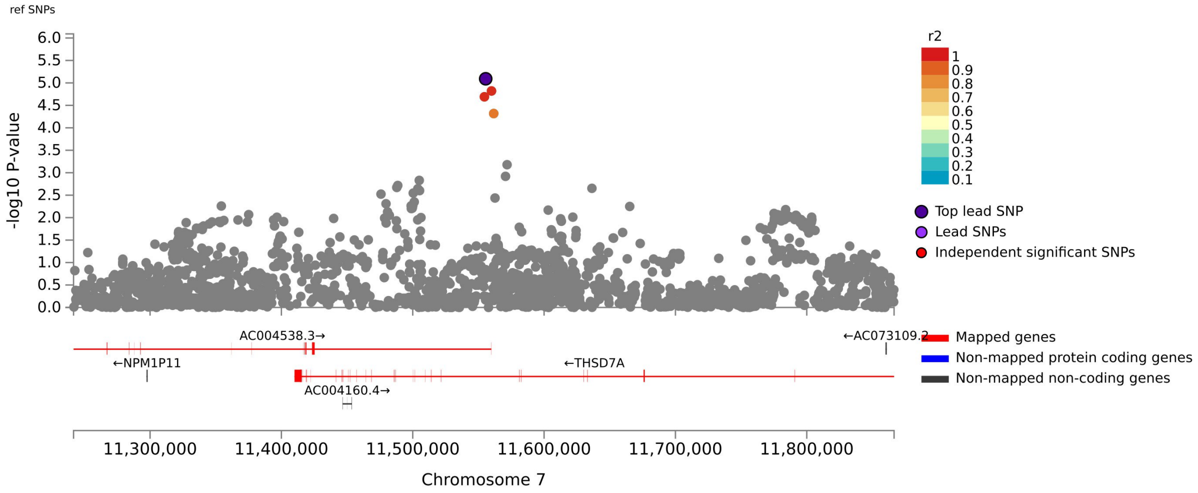

Suppl.Figure.S4. ***FUMA-GWAS analysis of variant-based meta-analysis: locus #4.*** Suggestive locus localized at chr14:40763557-40860432 (96,875 bps). It encompassed 12 variants with at least nominal significance ($p<0.05$), and implicated two genes by physical proximity and eQTL annotation in brain, blood and/or immune cells. The locus was led by the variant rs61989120 ($p=3x10^{-6}$, $Z=4.7$).

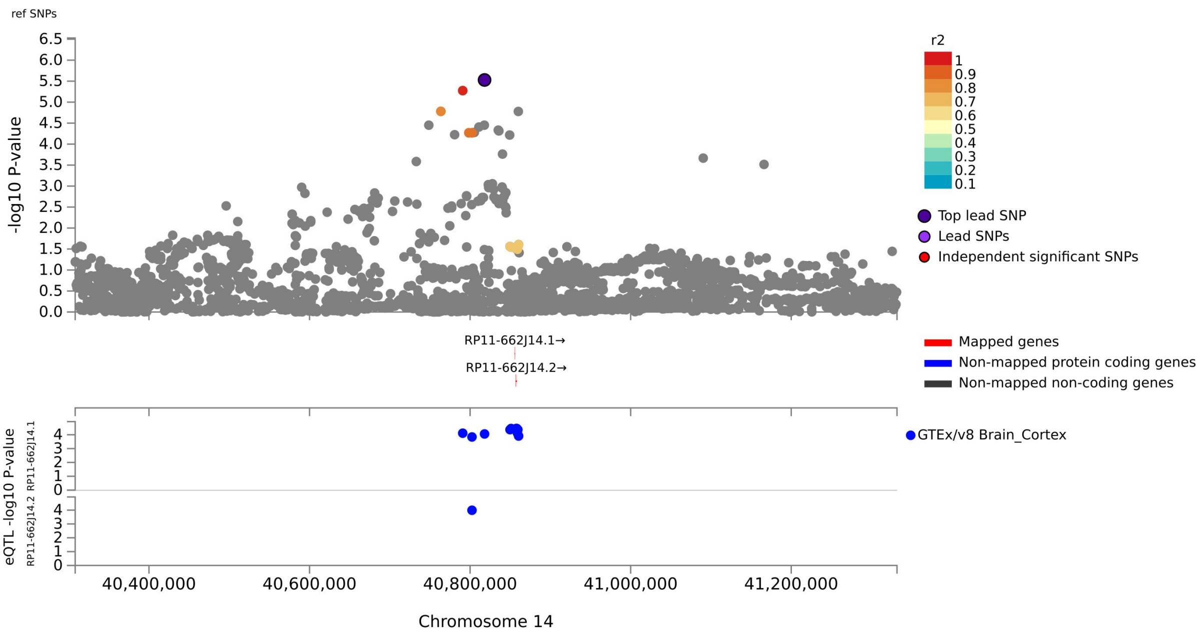

Suppl.Figure.S5. ***FUMA-GWAS analysis of variant-based meta-analysis: locus #5.*** Suggestive locus localized at chr17:19612489-19777031 (16,4542 bps). It encompassed five variants with at least nominal significance ($p<0.05$), and implicated nine genes by physical proximity and/or eQTL annotation in brain, blood and/or immune cells. The locus was led by the variant rs112155453 ($p=3.9 \times 10^{-6}$, $Z=4.6$).

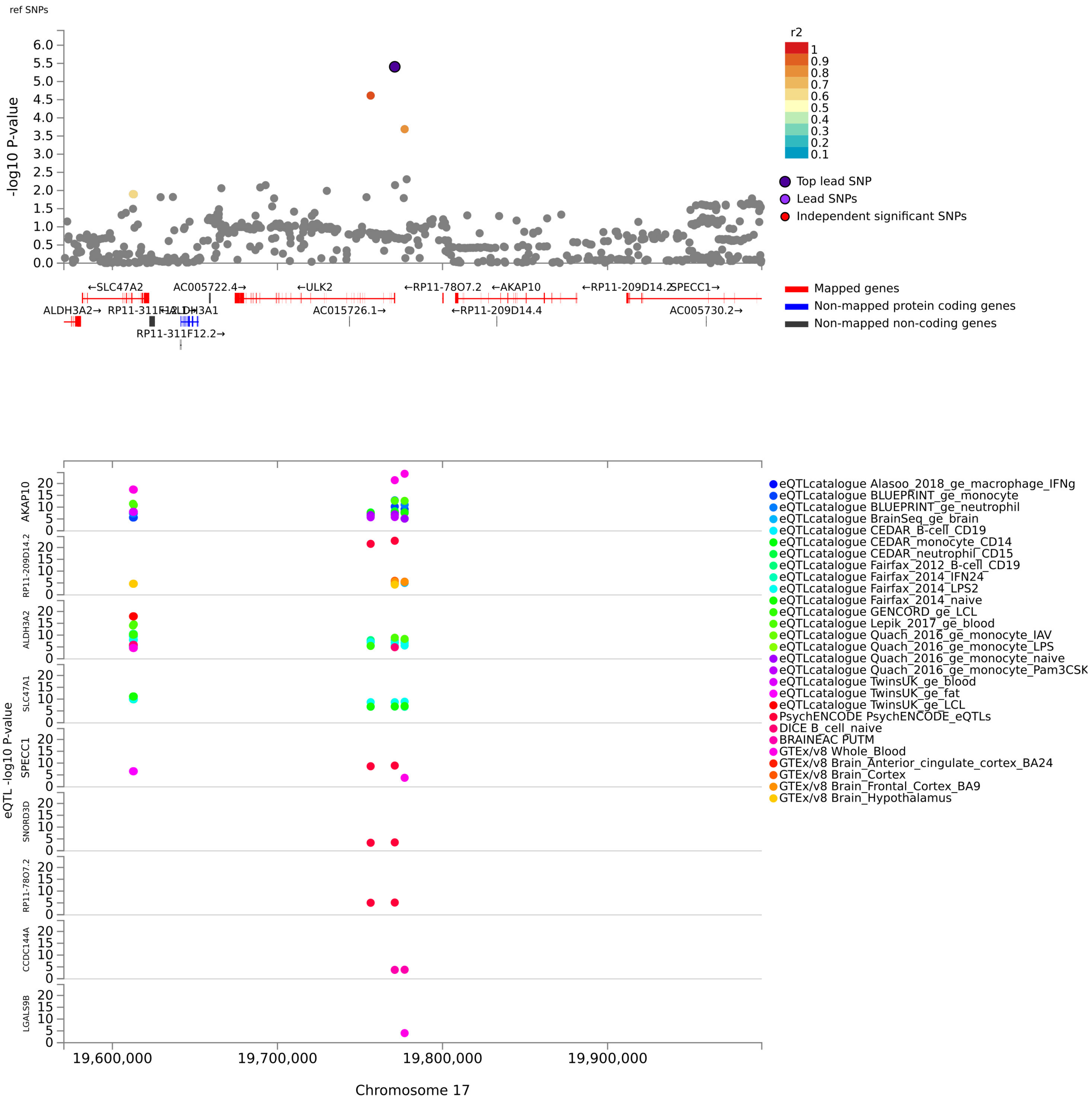

Supplement: Supplementary file 2 — Supplementary Figures [file 41380_2024_2688_MOESM2_ESM.pdf]
